# Seroprevalence and risk factors of HBV, HCV and HIV among hemodialysis patients: a multicenter cross-sectional study from Damascus Syria

**DOI:** 10.1186/s12879-024-09177-4

**Published:** 2024-03-06

**Authors:** Jehan Altinawe, Muhammad Eid Akkawi, Nihad Kharrat Helu, Qusai Hassan, Abdul-Hakim Nattouf

**Affiliations:** 1 Department of Pharmaceutics and Pharmaceutical Technology, Faculty of Pharmacy, Damascus University, Damascus, Syria; 2 Department of Pharmacy Practice, Faculty of Pharmacy, International Islamic University Malaysiam, Jalan Sultan Ahmad Shah, Bandar Indera Mahkota, Kuantan, Pahang 25200 Malaysia; 3 Department of Internal Medicine, Faculty of Medicine, Damascus University, Damascus, Syria

**Keywords:** Hemodialysis, Infection, Hepatitis, HBV, HCV, HIV, Chronic kidney disease, Damascus, Syria

## Abstract

**Objective:**

The aim of this study is to determine the prevalence rates of hepatitis B virus (HBV), hepatitis C virus (HCV), and human immunodeficiency virus (HIV) infections among hemodialysis (HD) patients as well as to identify associated risk factors.

**Methodology:**

A multicenter cross-sectional study involved patients who had been on HD for at least three months. The study was conducted at five HD centers in Damascus, Syria from August 2019 to September 2021. HBsAg, HCV-Ab and HIV (antibody/antigen) seropositivity were identified using the third generation ELISA technique. Patients’ information was extracted from their records and by face-to-face interview. Multiple logistic regression models were applied to identify risk factors associated with HBV or HCV seropositivity. The significance level was set at 5%.

**Results:**

A total of 637 patients were included in the study with a mean age (SD) of 50.5 (15.6) years and 56.7% of them were men. The dialytic age ranged from one to thirty years with a mean (SD) of 6.10 (5.6) years. The prevalence of positive hepatitis B surface antigen, anti-HCV, co-infection of HBV and HCV, and anti-HIV (antibody/antigen) were 3.2%, 22.1%, 0.7%, and 0%, respectively. After controlling for co-variables, hepatitis B vaccine was the only predictor of seropositivity of HBV (OR: 0.15, 95% CI: 0.057–0.393, *P* < 0.001), as it significantly protected against contracting HBV. On the other hand, the dialytic age (OR: 1.42, 95% CI: 1.12–1.94, *P* = 0.032) and the dialysis center were significant factors affecting the prevalence of HCV.

**Conclusions:**

The prevalence of HCV and HBV infections among HD patients in Damascus, Syria has decreased remarkably compared with the results from 2001. Nevertheless, it is still considered relatively high. Thus, there is an urgent need to strengthen the prevention and control measures for viral infection transmission in HD centers in Damascus.

## Introduction

Chronic kidney disease (CKD) has become one of the major public health problems worldwide. It is considered a progressive condition that affects more than 10% of the general population worldwide, numbering over 800 million people due to the increased prevalence of hypertension, diabetes mellitus, and other risk factors [1, 2]. Hemodialysis (HD) is the management option for patients with end-stage kidney disease (ESKD). As for any intervention, HD is associated with several adverse effects including contracting viral infections. This has a multitude of causes underlying both the patient and the HD procedure. These viral infections are a main cause of death following cardiovascular diseases in HD patients [3]. Hepatitis B virus (HBV), hepatitis C virus (HCV), and human immunodeficiency virus (HIV) infections are key factors for morbidity and mortality in patients on HD and also pose problems when handling CKD patients at dialysis centers [4, 5]. HCV has a significant global impact, with 58 million people chronically infected and about 1.5 million new infections occurring each year [6] which is responsible for more than one million deaths annually due to cirrhosis and liver hepatocellular carcinoma [7]. Likewise, HBV is an important health problem worldwide; it is estimated that about 350 million people are chronically infected with 250,000 deaths annually [8]. Issues in the Middle East such as wars and conflicts have contributed to many problems like difficulty of securing HIV prevention services, destruction of health care infrastructure, disruption of society to support networks, shortage of medical equipment, increased exposure to sexual violence, expansion of migration and displacement, and multiplication of the infection rate for acquired immunodeficiency syndrome (AIDS) and associated mortality [9].

Studies reported several risk factors related to the prevalence of HBV and HCV among general population including frequent visit to barbers, having multiple sexual partners, ear and nose piercings, dental surgeries, tattooing, circumcision, use of intravenous (IV) illicit drugs, recipient of blood products and undergoing invasive nosocomial procedures (e.g., endoscopy) [10, 11]. While the most commonly reported risk factors among HD patients were suppressed immunity (HD procedure affects both innate and acquired immunity), multiple blood transfusion events, invasive medical procedures, continued vascular exposure and sharing of infested patients dialysis machines and surroundings [3, 12]. Patients with hemophilia, IV drug addicts, and on long-term HD were found to have the highest HCV incidence [13]. Therefore, HD patients are at risk of viral infections compared to other vulnerable groups [14, 15]. In the Middle East countries, the average HCV infection rate among HD patients was 25.3% with low prevalence in Iran (12%), Turkey (23%), and in Iraq (20%), but high rate in Egypt (50%) [16]. The prevalence of HBV infection in dialysis centers in developed countries was ranged between 1.2% and 6.6% of the patients, while it is higher in developing countries (1.3–14.6%) [17].

The available studies in Syria on the prevalence of HVB, HCV, and HIV in HD patients are limited and outdated. In 2001, the prevalence of HCV among HD patients in Damascus ranged from 24.4 to 88.6% [14]. Data from Aleppo in 2009 showed prevalence rates of HBV and HCV of 52.9% and 54.4%, respectively [18]. The Syrian ministry of health has imposed the implementation of the standard global infection control measures which include isolation of infected patients, having dedicated dialysis machines for infected patients, wearing gloves and to be changed between patients, preparing injectable drugs in clean room, disinfection of HD machines both internally and externally, disinfection the surfaces of HD environment, using disposable items when possible and single use of dialyzers [19]. Therefore, there is a crucial need for an update on the prevalence rates of these common viral infections among Syrian HD patients. Although, a reduction in the prevalence of hepatitis is expected due to the updated infection control measures implemented in Syria, it is believed that this prevalence is still high compared with developed countries. The current study aimed to assess the prevalence of HVB, HCV, and HIV among HD patients from five dialysis centers in Damascus, Syria. Also, the study investigated risk factors associated with contracting these infections.

## Materials and methods

### Study design and population

This cross-sectional, multicenter study was conducted at five medical centers in Damascus, namely, Al-Assad hospital, Al-Mouassat hospital, the kidney hospital, Al-Zabadani hospital, and Hassan Al-Tahhan charitable dialysis center, from August 2019 to September 2021. Patients who had been on HD for the last three months were included in the study. Patients who were already diagnosed with HBV, HCV or HIV were excluded from the study. Patients who were not able to communicate due to any reason were also excluded.

### Sample size

The formula used to calculate the required sample size is [20]

$$ {\text{n}}^{\prime }=\frac{{\text{N}}{{\text{Z}}}^{2}{\text{P}}(1-{\text{P}})}{[{{\text{d}}}^{2}\left({\text{N}}-1\right)+{{\text{Z}}}^{2}{\text{P}}\left(1-{\text{P}}\right)]}$$

Where:

n′ = Sample size with finite population correction,

N = Population size = 20,000 based on a previous study [21].

Z = Z statistic for a level of confidence, which is 1.*96*.

*P =* Expected proportion of HD patients with HCV, (set 0.5) and.

d = Precision, which is considered as 0.*05.*

$$ \text{n}^{\prime }=\frac{20,000 \times {\left(1.96\right)}^{2} \times 0.5(1-0.5)}{[{\left(0.05\right)}^{2} \times 19,999 + {\left(1.96\right)}^{2} \times 0.5\left(1-0.5\right)]}=377 $$

The minimum required sample size to represent the HD population in Damascus, Syria was therefore 377 patients.

### Data collection

Demographic information and medical history including HBV vaccine, past and current diseases, surgery and endoscopy, transfusion of blood or one of its components, previous use of parenteral drugs, kidney transplantation, number of HD sessions per week, and HD duration were collected using the patients’ records as well as via a face-to-face personal interview. Convenience sampling technique was used. Patients who attended their scheduled HD session were recruited in the study as they fulfilled the inclusion criteria.

### Blood sample collection and examination

A sample of 5 mL blood was collected from each patient before starting HD session by tubes containing lithium heparin. Following 5–8 min of centrifugation (3000 RP), serum taken from each patient was frozen at −80 °C until the analysis. Later, the serum was tested for Hepatitis B surface antigen (HBsAg), HCV antibodies (HCV-Ab), and HIV antibody-antigen (HIV-Ag) using the third-generation enzyme-linked immunosorbent assay (ELISA-3). Testing for HCV antibodies was repeated again for positive results for confirmation. All viral tests were conducted according to the manufacturer’s instructions using commercially available assays (Biokit, S.A., Spain) in the laboratories of the University Blood Bank in Damascus, Syria.

### Statistical analysis

Analysis of the obtained results was performed using SPSS version 25 (IBM Corp., Armonk, NY, USA). Participants’ characteristics were represented using descriptive statistics. The Chi-square/Fisher exact test, Mann-Whitney U test and T-test were also used to find out the degree of association between the presence of HBsAg, HCV-Ab, or HIV (Ab/Ag) infection and potential risk factors. The significance level was set at 5%. Logistic regression models were applied to test for risk factors of having positive HBsAg, HCV-Ab, or HIV (Ab/Ag) while controlling for co-variables. Patients’ demographic characteristics and clinically important variables were included in the regression models. The Hosmer & Lemeshow test was used to check goodness of fit of the logistic regression models.

## Results

A total of 637 patients were included in the study. The mean age ± SD was 50.5 ± 15.6 years with men patients represent 56.7% (390) of the study sample. Hypertension was the most common comorbidity which was found in 84.1% of the patients followed by cardiovascular diseases (CVD) (38.3%) and type-2 diabetes mellitus (21%). The majority of the patients (70.5%) reported being vaccinated against HBV. More than half of the patients (68.3%) have undergone blood or blood component transfusion. The HD duration ranged from one to thirty years, with a mean of 6.10 ± 5.6 years (Table 1).

**Table 1:** Patients’ demographic and medical characteristics (n:637)

Variable	N (%)*
**Gender**	
Male	(56.7%) 361
Female	(43.3%) 276
Age (Mean ± SD)	50.5 ± 15.6
**Hemodialysis center**	
1	253 (39.7%)
2	149 (23.4%)
3	146 (23%)
4	76 (11.9%)
5	13 (2%)
Number of surgeries (Mean ± SD) [range]	2.75 ± 1.6 [0–11]
History of blood transfusion	435 (68.3%)
Number of blood transfusion events (Mean ± SD) [range]	3.40 ± 4.6 [0–36]
Hemodialysis duration (Mean ± SD) [range]	6.1 ± 5.6 [1–30]
**Hemodialysis sessions per week**	
1–2	570 (89.3%)
3	67 (10.5%)
History of dental procedure	(72.5%) 462
History of gastrointestinal endoscopy	(39.2%) 250
History of using parenteral medications	(35.8%) 228
HBV Vaccinated	(70.5%) 449
Kidney transplantation	(5.7%) 36
**Comorbidity**	
Hypertension	536 (84.1%)
Cardiovascular diseases	244 (38.3%)
Diabetes mellitus	134 (21%)

The overall prevalence of HBV was 3.2%, while it was 22.1% for HCV. Co-infection of hepatitis B with C was found in 0.7% of the study patients. No positive case of HIV was found among the patients. Table 2 shows the association between the prevalence of HBV or HCV and patients’ medical and demographic characteristics. The results show that patients who undergo HD sessions at the first center are more likely to be infected with HCV compared to the rest of the centers (*P* < 0.001). Patients who did not receive HBV vaccine were more likely to have hepatitis B compared to patients who received the vaccine (*P* < 0.001). It was also found that patients who have a history of blood/blood component transfusion or dental procedure were more likely to be positive to HCV compared to other patients (*P* < 0.001) (Table 2).

**Table 2:** Differences in the prevalence rates of HBV & HCV infection based on patients’ categorical variables (n:637)

Variable		Positive HBsAg N (%)	P-value	Positive HCV-Ab N (%)	P-value
Hemodialysis center	1	9 (3.6%)	0.06	105 (41.5%)	< 0.001
	2	5 (3.4%)		18 (12%)	
	3	5 (3.4%)		16 (11%)	
	4	0 (0%)		1 (1.3%)	
	5	0 (0%)		0 (0.0%)	
Had HBV Vaccine	Yes	7 (1.6%)	< 0.001	107 (23.8%)	0.977
	No	15 (8%)		45 (23.9)	
Having Diabetes	Yes	4 (3%)	0.738	22 (16.4%)	0.023
	No	18 (3.6%)		130 (25.8%)	
Having Hypertension	Yes	17 (3.2%)	0.369	121 (22.6%)	0.079
	No	5 (5%)		31 (30.7%)	
Having CVD	Yes	7 (2.9%)	0.524	57 (23.4%)	0.815
	No	15 (3.8%)		95 (24.2%)	
Had dental Procedures	Yes	16 (3.5%)	0.983	93 (20.1%)	< 0.001
	No	6 (3.4%)		59 (33.7%)	
Had GI endoscopy	Yes	12 (4.8%)	0.135	63 (25.2%)	0.524
	No	10 (2.6%)		89 (23%)	
Underwent blood transfusion	Yes	17 (3.9%)	0.357	123 (28.3%)	< 0.001
	No	5 (2.5%)		29 (14.4%)	
Previous use of parenteral drugs	Yes	7 (3.1%)	0.692	58 (25.4%)	0.486
	No	15 (3.7%)		94 (23%)	
Kidney transplantation	Yes	1 (2.8%)	0.819	10 (27.8%)	0.570
	No	21 (3.5%)		142 (23.6%)	
Number of hemodialysis sessions per week	2	19 (3.3%)	0.874	134 (23.6%)	0.570
	3	3 (4.5%)		18 (26.9%)	

Also, age, number of blood transfusion events, and dialytic age were significantly associated with contracting HCV infection (*P* < 0.001) (Table 3).

**Table 3:** Differences in the prevalence rates of HBV & HCV infection based on patients’ continuous variables

Variable	Positive HCV-Ab			Positive HBsAg		
	Yes	No	P value	Yes	No	P value
Age (mean ± SD)	47.18 ± 15.07	51.52 ± 15.6	0.006	50.73 ± 9.89	50.48 ± 15.73	0.857
Number of surgeries (mean ± SD)	2.59 ± 1.344	2.8 ± 1.632	0.384	3.05 ± 1.618	2.74 ± 1.56	0.289
Number of blood transfusion events (mean ± SD)	4.39 ± 4.71	3.09 ± 4.53	< 0.001	5.23 ± 6.488	3.34 ± 4.517	0.152
Hemodialysis duration (Dialytic age) (mean ± SD)	9.21 ± 7.44	5.13 ± 4.45	< 0.001	5.82 ± 5.72	6.11 ± 5.58	0.590

To correct for possible confounding factors, logistic regression models were applied for both HBV and HCV. The variables included in the models were basic demographic characteristics (age and gender), clinically important factors (history of blood transfusion, number of blood transfusion events, number of surgeries and history of dental procedure) and variables showed potential significance (*P* < 0.25) in the bivariate analysis (HD center, dialytic age and having DM for HCV regression model; HD center and HBV vaccine for HBV model). All assumptions required to apply the binomial regression were met before running the model. The applied logistic regression models showed that only hepatitis B vaccine significantly affects the prevalence of hepatitis B where it was a significant protector against contracting HBV (Table 4). On the other hand, the dialytic age and the dialysis center were significant factors affecting the prevalence of HCV (Table 5).

**Table 4:** Results of the binomial logistic regression model for positive HBsAg

Variable	Unstandardized coefficient (B)	P value	OR	95% CI
Gender (Male)	0.684	0.192	1.982	0.71–5.534
Age	0.01	0.618	1.01	0.971–1.051
Hepatitis B Vaccine	−1.898	**< 0.001**	0.15	0.057–0.393
History of blood transfusion (No)	0.328	0.614	1.388	0.389–4.953
Number of surgeries	−0.178	0.23	0.837	0.626–1.119
History of GI endoscopy (No)	0.754	0.124	2.126	0.814–5.552
Dental procedure (No)	0.302	0.57	1.352	0.478–3.822
Number of blood transfusion events	−0.04	0.982	0.988	0.336–2.906
Dialysis center (5)	–	0.915	–	–
Center 1	0.85	0.996	2.339	0.246–5.873
Center 2	0.79	0.999	2.203	0.483–6.789
Center 3	0.17	0.791	1.185	0.339–4.143
Center 4	0.569	0.326	1.767	0.567–5.508

OR: odds ratio, CI confidence interval, GI gastrointestinal

**Table 5:** Results of the binomial regression model for Positive HCV-Ab

Variable	Unstandardized coefficient (B)	P value	OR	95% CI
Gender (male)	−0.339	0.413	0.713	0.317–1.603
Age	−0.014	0.386	0.987	0.957–1.017
History of blood transfusion (No)	0.68	0.239	1.974	0.637–6.121
Number of surgeries	0.22	0.137	1.246	0.932–1.666
History of GI endoscopy (No)	0.349	0.437	1.418	0.588–3.416
Dental procedure (No)	−0.228	0.602	0.796	0.338–1.876
Number of blood transfusion events	0.013	0.791	1.013	0.922–1.113
Dialysis center (5)		**< 0.001**		
Center 4	1.314	0.680	3.721	0.24–7.82
Center 3	1.62	0.019	4.9	1.2–6.28
Center 2	1.83	0.001	6.23	2.033–9.578
Center 1	2.274	< 0.001	9.722	2.95–32.035
Having DM (No)	1.022	0.069	2.778	0.924–8.53
Dialytic age	0.133	**0.032**	1.422	1.12–1.94

OR: odds ratio, CI confidence interval, GI gastrointestinal, DM Diabetes Mellitus

## Discussion

Hepatitis B and C viruses represent a public health problem among HD patients and are considered a global health epidemic. There is a scarcity of information about the prevalence of hepatitis in Syria. In 2004 the HCV seroprevalence was estimated to be 2.8%, with a higher prevalence of HBV estimated as 5.6% of the Syrian population [22]. In 2014, much more blood units were positive for HBV (about 1.1%), compared to the general prevalence of HCV (about 0.4%) at Syrian blood banks [22].

The results of the current study showed that the prevalence rates of HBV, HCV and HBV-HCV co-infection among HD patients were 3.2%, 22.1% and 0.7%, respectively. The literature shows that the prevalence of HBV and HCV among HD patients significantly varies from country to country as well as from area to area of the world. It was reported that the prevalence of HCV among HD patients in developed and developing countries to be (1.4–28.3%) and (4.7–41.9%), respectively [23]. On the other hand, the prevalence of HBV among HD patients was estimated to be (0–6.6%) in developed countries [24]. Our results are similar to previous studies from other developing Arab countries where the prevalence rates of HBV and HCV were showed to be 3% and 21.7% respectively in Yemen [12] and about 20.9% of the overall HD patients were infected with HCV in Sudan [25]. However, the prevalence rates of HBV/HCV in our study were higher than those reported from neighboring countries such as Lebanon (1.6%:4.7%) [26], Iraq **(**3.2%/3.4%) [7], and Palestine (3.8%/7.4%) [5]. On contrary, our results showed lower HCV prevalence than other developing countries, such as Egypt (34.8%) [10] and Kosovo (53%) [27]. It is worth noting here that the current results demonstrate a major reduction in the prevalence of HCV/HBV among HD patients in Syria. Two decades ago, the prevalence of HCV/HBV among HD patients in Damascus was reported to be 48.9%/15.8% [14]. Also, the prevalence rates of HBV and HCV from HD centers in Aleppo in 2009 were 52.9% and 54.4%, respectively [18]. Our finding regarding the co-infection of hepatitis C and hepatitis B together was lower compared to the other regional countries, such as Iran (4.49%) [4], Yemen [12], Palestine (1.6%) [15], and Morocco (2.9%) [28].

The reason for the different prevalence rates between these countries may be attributed to: the rigid application of infection control and effective global measures to prevent the out-spread of blood-transmitted viruses, the difference in the local prevalence rates, and the health care system settings in each country [2, 29]. This also explains the reported significant reduction of the prevalence of HBV and HCV compared to the old Syrian studies. It can clearly be attributed to an improvement in clinical practices in terms of better patient monitoring and application of virus detection tests, which became more sensitive and specific (the third generation of the anti-HCV ELISA). It is always necessary to establish strict adherence rules consistent with the global guidelines for prevention and control of viral infection spread among HD patients [30]. It is worth mentioning here that some violations to the infection control protocol were observed sometimes during this study in some HD centers. Such violations include: not wearing gloves or not changing them when handling different patients, insufficient sterilization of the surfaces of the dialysis environment (beds, tables, and carts) and no clean area designated for preparing parenteral drugs. Also, the guidelines issued by the Syrian ministry of health stipulate that serological tests to investigate HBV, HCV and HIV must be performed every 6 months for HD patients. Nonetheless, this was not practiced in some centers due to the lack of laboratory kits which cannot be afforded by these centers. On the other hand, all centers were compliant in terms of applying efficient chemical or thermal methods for sterilizing dialysis machines internally and externally, not reusing dialyzers, dedicating dialysis devices for infected patients and locating infected patients with HCV or HBV in isolated halls, except for one center where there was juxtaposition between HCV negative and positive patients.

No positive case of HIV was recorded in the study sample. This finding is identical to the results reported from neighboring countries like Iraq [3] and Palestine [31]. The reason could be due to cultural, social, and religious beliefs where having multiple sexual affairs is prohibited.

The only factor significantly associated with the prevalence of HBV was hepatitis B vaccine. After controlling for co-variables, it was found that unvaccinated HD patients had 6.6 folds the risk of having HBV compared with vaccinated patients. HBV vaccine was consistently reported to be an effective protective factor against contracting HBV infection for HD patients, in Syria [18, 32] and other countries [33]. This also explains the low prevalence rate of HBV compared to HCV found in our study. In Syria, the HBV vaccination protocol obligates all patients with ESRD to take three doses of the HBV vaccine before reaching the HD stage. Other predictors of positive HBV reported in the literature such as history of blood transfusion [15, 34] and dialytic age [32, 34] did not affect the prevalence of HBV in our study. However, other studies support our findings where none of these factors were significant predictors [36].

For HCV prevalence, the current study revealed that longer dialysis duration (dialytic age) significantly increased the risk of contracting HCV. Similar finding was reported from Syria [14, 32] neighboring countries [15, 36] and many other countries worldwide [34, 37–40]. The impact of dialytic age is consistent with the nosocomial transmission of HCV infection, as epidemiological and molecular studies showed the role of the dialysis environment in the spread of HCV infection among HD patients. This association may be due to the fact that dialysis itself is an invasive procedure that requires prolonged and repeated access to blood vessels and thus exposure to potentially contaminated equipment shared by HCV-infected patients with lack of adherence to transmission prevention measures [4, 41]. A clinical virological study from the Middle East found that HCV RNA presents in the handwashing of nurses responsible for dialysis of HCV positive and negative patients [42]. Also, the current study has proven a difference in the prevalence of HCV among the HD centers. Even after controlling for other variables, HD center was still a significant predictor of having positive HCV. Patients who were attending centers 1, 2 and 3 had higher risk of contracting HCV infection. Differences may be due to violations or noncompliance with infection control procedures at individual centers or because of the deficiency in hospital facilities. Compared to centers 4 and 5, the three other centers serve high number of HD patients monthly with limited number of medical personnel and shortage (due to the current situation in Syria) in the equipment and consumable materials that are necessary for implementation of infection preventive measures. On top of that, there was no routine audit on the compliance of the staff with the nosocomial infection control guidelines in the study centers. In fact, a detailed assessment of each HD center, especially in those with the highest prevalence of HBV or HCV, is crucially needed to evaluate the reasons for the differences between the HD centers. Similar findings were reported previously from Syria [14, 18] and the neighboring countries; Palestine [15] and Lebanon [26].

Other risk factors reported by other studies such as history of blood transfusion [15, 34], history of surgery [25, 36], and male sex [12] were not significantly associated with HCV prevalence among our study patients. However, the risk factors for HCV infection among HD patients varies notably between countries. Several other studies agree with our results as none of the above factors were significant predictors of HCV contamination among their HD patients [28, 32, 35, 40]. The difference in the results could be attributed mainly to the degree of adherence to the standard infection preventive measures and the new methods/technologies used [35]. For instance, blood transfusion and surgical intervention were historically major risk for transmission of hepatitis infection [43]. However, this risk has been dramatically reduced with the implementation of new protocols that obligate blood screening for the donors as well as applying strict sterilization and hygiene procedures for operation theaters, equipment and personnel [44, 45].

There are some limitations of the current study. First, the HCV seroprevalence was not confirmed through the detection of HCV RNA by PCR in blood samples. Likewise, hepatitis B core antibodies and HBV DNA by PCR were not applied due to financial difficulties. Second, we cannot confirm that the HBV or HCV infection was contracted after starting HD, although routine screening for both viruses is required for all patients before being enrolled in one of the HD centers.

## Conclusions

The current study showed that the prevalence of HCV and HBV infections among HD patients in Damascus, Syria has decreased from (48.9%–15.8%) in 2001 to (22.1%–3.2%) in 2021. Nevertheless, it is still considered relatively high compared with neighboring countries, and it remains a serious regional problem. Therefore, strict adherence to infection preventive measures will prevent and reduce the spread of blood-borne pathogens, including HBV and HCV. Hepatitis B vaccine was found to be an effective protector of HBV infection among this population. On the other hand, the duration of being on HD and the HD center were significantly associated with HCV infection. This emphasizes again the crucial importance of following the standard preventive measures to prevent viral infection spread among HD patients. An appropriate screening program for HBV and HCV in HD centers is highly recommended and positive patients might be isolated to limit the spread of infections. Routine anti-HCV antibody and HBsAg testing is recommended when patients become eligible for HD and when patients are transferred from one facility to another. HBV vaccination should be offered to all potentially infected patients and healthcare personnel. Educational campaigns for both health care providers and patients regarding viral infections at HD centers could also help in reducing viral infection transmission among this population.

## Data Availability

Data are available upon request from the corresponding authors.
